# Cyclin D1 G870A Polymorphism and Risk of Nasopharyngeal Carcinoma: A Meta-Analysis

**DOI:** 10.1155/2013/689048

**Published:** 2013-10-03

**Authors:** Meng Li, Weijian Dai, Huanqin Zhou

**Affiliations:** ^1^Department of Laboratory Medicine, Zhejiang Hospital, Hangzhou 310013, China; ^2^Zhejiang Provincial Key Lab of Geriatrics, Hangzhou 310013, China; ^3^Department of Laboratory Medicine, The First People's Hospital of Hangzhou, Hangzhou 310006, China

## Abstract

Recently, there have been a number of studies on the association between cyclin D1 G870A polymorphism and nasopharyngeal carcinoma risk. However, the results of previous reports remain controversial and ambiguous. Thus, we performed a meta-analysis to explore more precisely the association between cyclin D1 G870A polymorphism and the risk of nasopharyngeal carcinoma. No significant association was found between cyclin D1 G870A polymorphism and nasopharyngeal carcinoma risk in total population analysis. In the subgroup meta-analysis by ethnicity, a negative association was shown in Caucasian subgroup, and no significant association in any genetic models among Asians was observed. In summary, positive results have been shown on the search for polymorphic variants influencing the risk of NPC. This meta-analysis provides evidence of the association between CCND1 G870A polymorphism and NPC risk, supporting the hypothesis that CCND1 870A allele probably acts as an important NPC protective factor in Caucasians but not in Asians. Since the results of our meta-analysis are preliminary and may be biased by the relatively small number of subjects, they still need to be validated by well-designed studies using larger samples in the future.

## 1. Introduction

Nasopharyngeal carcinoma (NPC) is a malignant epithelial cancer with a strikingly geographic and ethnic distribution. The incidence of NPC is higher in Southeast Asia and Africa, but lower among Caucasians in North America and Europe. Epidemiological studies and experimental researches have implicated genetic susceptibility, Epstein-Barr viral (EBV) infection, and environmental factors in the specific and multifactorial etiology of NPC [1]. In spite of many individuals being exposed to EBV infection and environmental risk factors including extensive tobacco and alcohol consumption, only a small population can be clinically diagnosed with NPC, which suggests that individual genetic susceptibility might play a more important role in the carcinogenic mechanisms of NPC. However, the precise genetic alterations during NPC development are still unclear.

Cyclin D1, encoded by *CCND1* gene, plays a critical role in the transition from G1 to S phase of the cell cycle during cell division. *CCND1* amplification and protein overexpression were detected (described) in NPC patients [2]. The activation of cyclin D1 participates in tumorigenesis [3], local tumor recurrence, and poor prognosis of NPC [2]. *CCND1* G870A (rs603965) polymorphism is common in a variety of human tumors, including breast cancer, lung cancer, gastric cancer, gynecological cancer, blood-related cancer, and colorectal cancer (NPC) [4, 5]. Although *CCND1* G870A polymorphism is a silent mutation (Pro241Pro), 870A allele results in an alternatively spliced transcript of *CCND1* (transcript b), which has been shown to have a longer half-life than the G allele (transcript a) encoded protein. It suggests that *CCND1* 870A allele is more likely to contribute to cancer development through promoting the transition between G1 and S phases [6]. To date, although a few studies have linked the *CCND1* G870A polymorphism to the increased NPC risk, the results remain controversial.

Considering a single study may be insufficient to identify the effect of CCND1 G870A polymorphism on NPC, and the published results have been controversial. We therefore performed a meta-analysis to assess the association between CCND1 G870A polymorphism and NPC susceptibility.

## 2. Methods

### 2.1. The Literature Search Strategy

We searched the literature databases including PubMed, ISI Web of Science, Chinese National Knowledge Infrastructure (CNKI), and Google Scholar (up to November 08, 2012). The search strategy for identifying all possible studies involved usage of the following keywords: “cyclin D1 or CCND1” and “polymorphism or variant or genotype or SNP” and “nasopharyngeal carcinoma or nasopharyngeal cancer or squamous cell cancer of the head and neck or head and neck cancer”. All related studies without language limitation were included. The reference lists of the additional articles reporting the association between CCND1 G870A polymorphism and NPC were hand searched.

### 2.2. Eligible Studies and Data Extraction

All the studies included in the meta-analysis met the following inclusion criteria: (1) original papers associated cyclin D1 G870A polymorphism with NPC; (2) case control or cohort design was used, and (3) genotype distribution information, odds ratio (OR) with its 95% confidence interval (CIs), and *P* value were provided. The major exclusion criteria were (1) duplicate data, (2) case-only studies, (3) review articles, and (4) studies with association between cyclin D1 G870A polymorphism and head and neck cancer, which just described the number and anatomical distribution of tumors without specifically showing the genotype distribution information of each tumor, including NPC.

Data extraction for compliance with the inclusion/exclusion criteria was performed independently by two reviewers. Disagreements were resolved by further discussion among all authors. For each included study, the following information was extracted according to a fixed protocol: (1) name of the first author; (2) year of publication; (3) country; (4) ethnicity; (5) genotyping method; (6) genotype distribution in cases and controls; (7) *P* value for Hardy-Weinberg equilibrium (HWE) test in controls.

### 2.3. Statistical Analysis

The association between cyclin D1 G870A polymorphism and NPC was estimated by calculating pooled odds ratio (OR) and 95% confidence interval (CI) under a homozygote comparison model (AA versus GG), a heterozygote comparison model (GA versus GG), and a dominant model (AA + GA versus GG), respectively. The significance of pooled OR was determined by *Z* test. Cochran's chi-square-based Q statistic test was performed to evaluate the possible heterogeneity among individual studies. Pooled ORs were calculated according to a fixed model (DerSimonian-Laird method) or a random model (Mantel-Haenszel method) in the absence (*P* > 0.10) or presence (*P* ≤ 0.10) of heterogeneity. Heterogeneity was explored using the subgroup analysis of ethnicity (Asians and Caucasians). Publication bias was assessed statistically by Egger's test and Begger's test. The Hardy-Weinberg equilibrium (HWE) was determined using the chi-square test in control groups. All the data analyses were performed using STATA software version 11.0 (StataCorp LP, College Station, Texas, USA). All *P* values were calculated with two-sided analysis, and a *P* value less than 0.05 was considered as statistical significance.

## 3. Results

### 3.1. Study Selection

**Figure 1:**
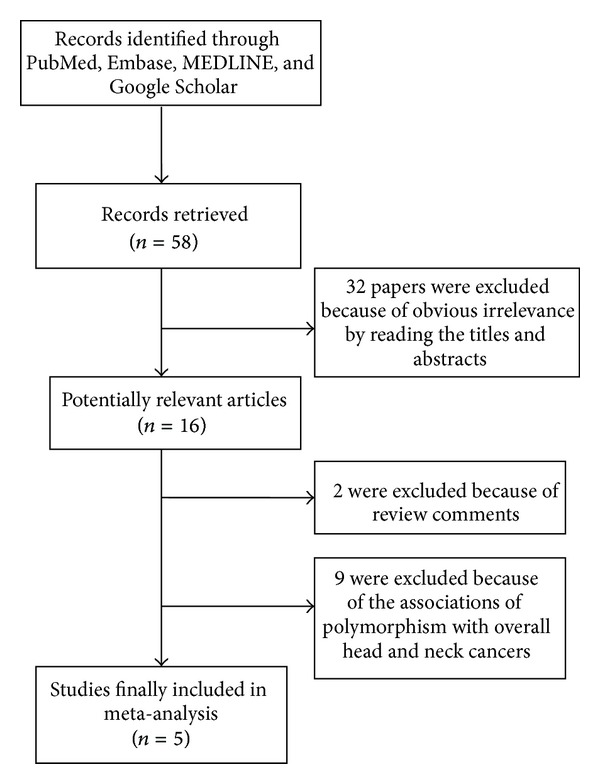
Flow chart of study selection based on the inclusion and exclusion criteria.

As shown in Figure 1, through the literature search, we have identified 58 potentially relevant papers. After a careful review, 32 papers were excluded because of obvious irrelevance by evaluating the contents of abstracts. In addition, two reviews [5, 7] and nine papers [8–16] which assessed the association between polymorphism and overall head and neck cancers were excluded. Finally, five studies (including 679 cases and 973 controls) studying the cyclin D1 G870A polymorphisms [17–21] met the inclusion criteria and were selected for the meta-analysis.

### 3.2. Characteristics of the Studies

Characteristics of the studies included in this meta-analysis were presented in Table 1. All studies were case-controlled. Of these 5 studies, 4 used polymerase chain reaction-restriction fragment length polymorphism (PCR-RFLP) and 1 used denaturing high-performance liquid chromatography (DHPLC). All studies were carried out in the mainland, Taiwan of China, and Portugal. Three studies were on Asians, and two studies were on Caucasians. Studies being carried out in the mainland and Taiwan of China were grouped to the Asian subgroup, while the others were grouped to the Caucasian subgroup. The distribution of genotypes in the controls was consistent with Hardy-Weinberg equilibrium (*P* > 0.05) in all studies except for three studies (Catarino et al. [17], *P* = 0.037; Shih et al. [20], *P* = 0.007; Catarino et al. [18], *P* = 0.047).

### 3.3. Quantitative Data Synthesis

**Figure 2:**
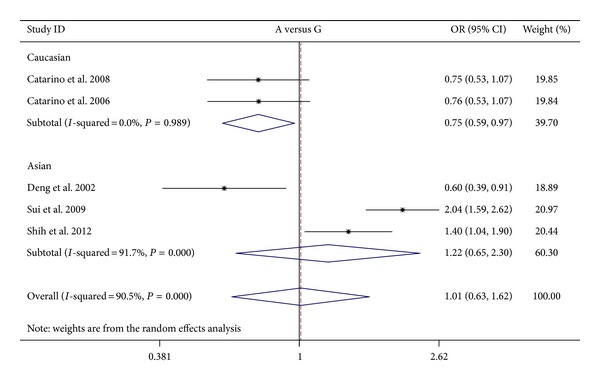
Forest plots of cyclin D1 G870A polymorphism in nasopharyngeal carcinoma versus normal control and subgroup analyses for A genotype compared with G genotype. The squares and horizontal lines correspond to the study specific OR and 95% CI. The area of the squares reflects the weight (inverse of the variance). The diamond represents the summary OR and 95% CI. OR: odds ratio.

**Figure 3:**
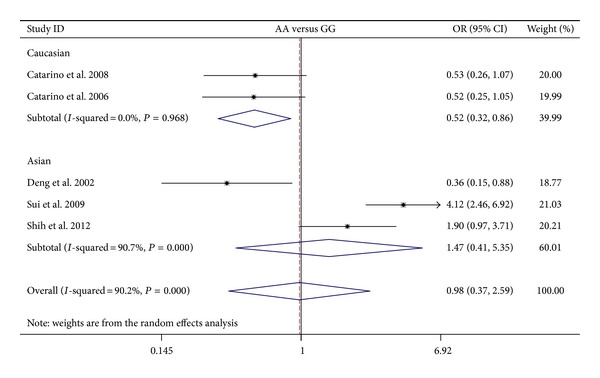
Forest plots of cyclin D1 G870A polymorphism in nasopharyngeal carcinoma versus normal control and subgroup analyses for AA genotype compared with GG genotype.

**Figure 4:**
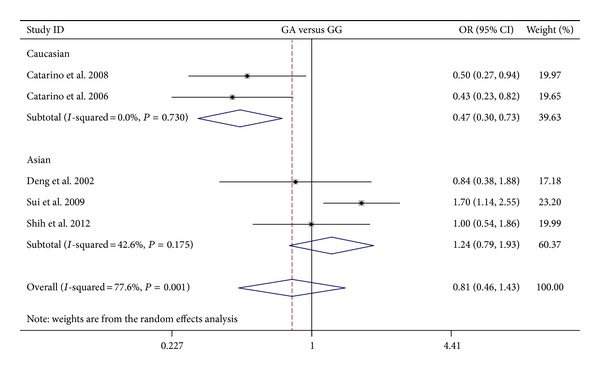
Forest plots of cyclin D1 G870A polymorphism in nasopharyngeal carcinoma versus normal control and subgroup analyses for GA genotype compared with GG genotype.

**Figure 5:**
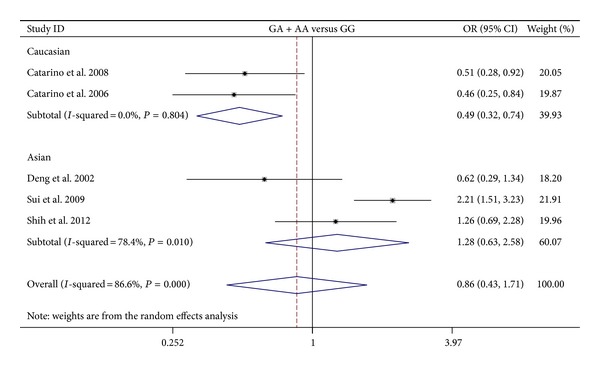
Forest plots of cyclin D1 G870A polymorphism in nasopharyngeal carcinoma versus normal control and subgroup analyses for AA + GA genotype compared with GG genotype.

The results on the association between cyclin D1 G870A polymorphism and NPC risk and the heterogeneity test were shown in Table 2. The combined results based on all studies showed that the variant genotypes were not associated with the increased NPC risk in different genetic models (OR = 1.010, 95% CI = 0.628–1.622 for A versus G, *P* = 0.969; OR = 0.976, 95% CI = 0.368–2.592 for homozygote comparison model AA versus GG, *P* = 0.961; OR = 0.811, 95% CI = 0.460–1.429 for heterozygote comparison model GA versus GG, *P* = 0.469; OR = 0.856, 95% CI = 0.430–1.707 for dominant model GA + AA versus GG, *P* = 0.660) (Figures 2, 3, 4, and 5). In the subgroup analysis by ethnicity, the results revealed a significant association between the cyclin D1 G870A polymorphism and NPC in Caucasian population (A versus G: OR = 0.754, 95% CI = 0.589–0.967, *P* = 0.026, *P*_het_ = 0.989; homozygote comparison model AA versus GG: OR = 0.524, 95% CI = 0.317–0.865, *P* = 0.011, *P*_het_ = 0.968; heterozygote comparison model GA versus GG: OR = 0.467, 95% CI = 0.299–0.730, *P* = 0.001, *P*_het_ = 0.730; dominant model GA + AA versus GG: OR = 0.487, 95% CI = 0.319–0.741, *P* = 0.001, *P*_het_ = 0.804). In contrast, no such significant association in any genetic models was observed in Asians (A versus G: OR = 1.221, 95% CI = 0.647–2.304, *P* = 0.538; homozygote comparison model AA versus GG: OR = 1.475, 95% CI = 0.407–5.345, *P* = 0.554; heterozygote comparison model GA versus GG: OR = 1.236, 95% CI = 0.791–1.913, *P* = 0.554; dominant model GA + AA versus GG: OR = 1.277, 95% CI = 0.631–2.584, *P* = 0.497).

### 3.4. Tests of Heterogeneity

Statistically significant heterogeneity was observed in trials using the following analyses with Q statistic tests (A versus G: *P* = 0.000, *I* ^2^ = 90.5%; homozygote comparison model AA versus GG: *P* = 0.000, *I* ^2^ = 90.2%; heterozygote comparison model GA versus GG: *P* = 0.001, *I* ^2^ = 77.6%; dominant model GA + AA versus GG: *P* = 0.000, *I* ^2^ = 86.6%) (Table 2) and employing the random-effects model.

### 3.5. Publication Bias

Egger's test and Beggar's test were performed to assess publication bias. Analysis using the Egger weighted regression method did not indicate publication bias for two of the four genetic models (heterozygote comparison model GA versus GG: *P* = 0.143; dominant model GA + AA versus GG: *P* = 0.082), but indicated evidence for publication bias for the other two genetic models (A versus G: *P* = 0.007; homozygote comparison model AA versus GG: *P* = 0.027). Beggar's rank correlation showed no evidence for publication bias for three of the four genetic models (A versus G: *P* = 0.086; heterozygote comparison model GA versus GG: *P* = 0.221; dominant model GA + AA versus GG: *P* = 0.462) but indicated publication bias for the homozygote comparison model AA versus GG (*P* = 0.027) (Table 3).

## 4. Discussion

Cell cycle regulation plays an important role in the development of cancer by influencing cell proliferation, differentiation, and apoptosis [22]. The *CCND1* gene encodes a key cell cycle regulatory protein, cyclin D1, which regulates transition from G1 to S phase during cell division. Cyclin D1 has been recognized as a promising biomarker for predicting tumor behavior [23]. In recent years, the common functional polymorphism, G870A in the gene cyclinD1, has been widely studied as a possible low-penetrant susceptibility allele for a variety of cancers. A number of studies found that the G allele seems to be a protective factor in hepatocellular carcinoma [24], laryngeal [25], breast [26], colorectal [27, 28], and bladder tumors [29]. But several controversial findings reported that the G allele was a risky factor for oral [30] and colorectal cancer [31] or was not associated with various types of cancer [32–36]. Since conflicting results among studies or ethnic groups have been reported, it is necessary to make a quantitative and summarized evaluation of possible association between cyclin D1 G870A polymorphism and risk of cancer.

Deng et al. found that cyclin D1 G870A polymorphism was associated with the susceptibility to NPC, because the GG and AG genotypes in NPC patients were significantly higher than those in normal controls [19]. But Shih et al. reported that the G allele of *CCND1 *G870A seemed to be a protective factor for NPC in Taiwan of China [20]. Catarino et al. also reported that individuals carrying the CCND1 GG genotype had increased risk for the development of NPC [18]. Therefore, it is worthy to make a meta-analysis to evaluate the interrelationship between cyclin D1 G870A polymorphism and NPC. The current meta-analysis summarized the results from 5 case-controlled studies on the association between the CCND1 G870A polymorphism and NPC risk. A total of 679 cases and 973 controls were included. Although we found no significant risk of NPC associated with the CCND1 G870A polymorphism based on total population, significant association was found in Caucasian population in the subgroup analysis by ethnicity.

In the subgroup meta-analysis based on ethnicity, compared with G allele, a significantly decreased risk of NPC was associated with A allele; compared with GG genotype, a significantly decreased risk of NPC was associated with AA genotype, GA genotype, and combined AA/GA genotype in the Caucasian subgroup. Further investigations on a larger scale on Caucasian population may be needed to confirm this result. In the Asian subgroup, no significant association was found in the different genetic models. Our results indicate that ethnicity may be a critical factor affecting effects of the polymorphic alleles on susceptibility to NPC.

Despite these advantages, some limitations in the current meta-analysis should be considered with caution. Firstly, the controls were not uniformly defined. Although most control groups were selected from healthy populations, some might have a benign disease. Therefore, there is a lack of proper matching, and the results are based on unadjusted estimates. Secondly, the analysis did not consider gene-gene and gene-environment interactions due to the lack of sufficient data. Thirdly, environmental and lifestyle-related factors may influence the results of this analysis. A more precise analysis with individual data available might be considered, which could allow an adjustment estimate by sex, age, body weight, and lifestyle such as smoking and alcohol drinking. Fourthly, there is evidence of publication bias in the formal evaluation used in this study. The results of our meta-analysis may be biased by the relatively small number of subjects. Therefore, our conclusion still needs to be validated by well-designed studies with larger sample size in the future. Finally, and most importantly, whether the CCND1 G870A polymorphism is independently predictive of cancer risk remains controversial.

In summary, positive results have been shown in the search for polymorphic variants influencing the risk of NPC. This meta-analysis provides evidence of the association between CCND1 G870A polymorphism and NPC risk, supporting the hypothesis that CCND1 870A allele probably acts as an important NPC protective factor in Caucasians, but not in Asians.

## Figures and Tables

**Table 1:** Characteristics of the studies included in the meta-analysis.

First author	Year	Country	Ethnicity	Genotyping method	Genotype distribution						*P* for HWE^a^	References
					Case			Control				
					GG	GA	AA	GG	GA	AA		
Catarino	2008	Portugal	European	PCR-RFLP	23	40	21	40	138	69	0.036	[17]
Catarino	2006	Portugal	European	PCR-RFLP	26	42	26	28	105	54	0.047	[18]
Deng	2002	China (mainland)	Asian	DHPLC	19	48	17	14	42	35	0.811	[19]
Shih	2012	China (Taiwan)	Asian	PCR-RFLP	23	86	67	28	105	43	0.007	[20]
Sui	2009	China (mainland)	Asian	PCR-RFLP	60	110	71	115	124	33	0.962	[21]

^a^HWE in controls.

**Table 2:** Meta-analysis of the association between cyclin D1 G807A polymorphism and nasopharyngeal cancer risk.

Comparisons	Odds ratio	95% confidence interval	*P* value	Heterogeneity		Effects model
				*I* ^2^	*P* value	
A versus G	1.010	0.628–1.622	0.969	90.5%	0.000	Random
Asians	1.221	0.647–2.304	0.538	91.7%	0.000	
Caucasians	0.754	0.589–0.967	0.026	0.0%	0.989	
AA versus GG	0.976	0.368–2.592	0.961	90.2%	0.000	Random
Asians	1.475	0.407–5.345	0.554	90.7%	0.000	
Caucasians	0.524	0.317–0.865	0.011	0.0%	0.968	
GA versus GG	0.811	0.460–1.429	0.469	77.6%	0.001	Random
Asians	1.236	0.791–1.913	0.353	42.6%	0.175	
Caucasians	0.467	0.299–0.730	0.001	0.0%	0.730	
GA + AA versus GG	0.856	0.430–1.707	0.660	86.6%	0.000	Random
Asians	1.277	0.631–2.584	0.497	78.4%	0.010	
Caucasians	0.487	0.319–0.741	0.001	0.0%	0.804	

**Table 3:** Publication bias test for cyclin D1 G807A polymorphism.

Comparisons	Egger's test			Beggar's test *P* value
	Coefficient	*P* value	95% CI	
A versus G	−15.91	0.007	−23.44–−8.39	0.086
AA versus GG	−14.52	0.037	−27.36–−1.68	0.027
GA versus GG	−6.03	0.143	−15.77–3.71	0.221
GA + AA versus GG	−8.39	0.082	−18.76–1.96	0.462
